# Ultrasonographic screening of urinary schistosomiasis infected patients in Agulu community, Anambra state, southeast Nigeria

**DOI:** 10.1186/1755-7682-2-34

**Published:** 2009-10-28

**Authors:** Chinyelu A Ekwunife, Fabian C Okafor, Obioma C Nwaorgu

**Affiliations:** 1 Department of Parasitology and Entomology, Nnamdi Azikiwe University Awka, Anambra State, Nigeria; 2 Dept. of Zoology, University of Nigeria, Nsukka, Enugu State, Nigeria

## Abstract

**Background:**

The pathology of *Schistosoma haematobium *infection in 60 infected primary school children in Agulu community, Anambra State, southeast Nigeria, with over 50 ova/10 ml urine was assessed.

**Methods:**

The ultrasonographic examination was done using a sector scanner with convex probe. World Health Organisation method was used for classification and scoring of lesions. T-test and Coefficient of determination were used in analysis.

**Results:**

The pathologic effects due to *S. haematobuim *identified among the study group included irregularity of the bladder wall (25%), thickening of the bladder wall (10%) and massing of the bladder wall (3.3%). About 4(6.7%) and 1(1.7%) of the patients had the right pelvis and left pelvis of their kidney moderately dilated respectively. Identified bladder wall lesions had 69 scores while kidney dilation had 30 scores. The number of individuals with lesions correlated with intensity of infection. Male pupils (65.2%) had more lesions than females (34.8%). The difference observed in lesion distribution among males and females was found to be significant (df = 6, p < 0.05). All bladder and kidney lesions responded favorably to treatment with praziquantel (40 mg/kg-body weight).

**Conclusion:**

Health education campaign including showing the community members evidence of damages to the organs (from the ultrasound pictures) will go a long way in the control and prevention of the disease in this community.

## Background

Schistosomiasis due to *S. haematobium *is an important public health problem in Africa and the Middle East. The infection causes considerable morbidity in most affected individuals. The adult worms live mainly in the venous plexus of the urinary bladder and morbidity is caused by egg deposition in and around the urinary tract causing inflammation and lesions. *S. haematobium *related pathology is found mainly in the urinary bladder, the ureters and the kidneys. Presently schistosomiasis control programmes are targeted at morbidity reduction in populations [1]. However, most of the widely used methods for assessing the success of interventions involve parameters like egg-counts and biopsies which measure the level of infection but do not provide direct evidence about pathological changes especially as regards to kidneys and other related organs among community members. Planning of effective interventions should involve reduction in damage to internal organs. The change from transmission control to morbidity control was initiated by the development of safer and more effective drugs and simple diagnostic techniques [2]. This requires knowledge of what changes occur in various organs, how fast and how far they can be reversed by treatment and how soon they appear after reinfection. Ultrasound can make a valuable contribution to the monitoring of control programmes, and the data collected should enable informed decisions to be made about where resources can best be invested in measures to reduce morbidity. Therefore in the present study, ultrasound was used to asses the extent of damage to the bladder and kidney by *S. haematobium *among primary school children in Agulu community a urinary schistosome endemic community in southeast Nigeria. Also the rate at which the lesions due to *S. haematobium *infection could be reversed through treatment with praziquantel assessed.

## Materials and methods

Sixty pupils, from primary schools in Agulu community, Anambra State South Eastern Nigeria, who excreted over 50 egg/10 ml urine, were involved in this study. With consent and co-operation of the pupils, parents, head teachers and teachers, these children were transported to a private hospital in Onitsha, a commercial city in Anambra State for ultrasound studies. Twelve children were transported to Onitsha every week for this purpose during the study period. An Aloka SSD 500, convex probe (5 mHz) sector scanner was used for the assessment of the urinary bladder and kidney of these pupil.

### Preparation and viewing

Thirty minutes to an hour prior to their examination, each child received a cup of water. This is to ensure that their bladder was adequately filled. The bladder was viewed transversely by placing the probe above the pubic symphysis at the maximal cross sectional diameter of the bladder with a view of the distal part of the ureters. Wall irregularity with thickening up to 5 mm is recorded as irregularity. The left and right kidneys were also viewed laterally. Dilation was measured as the largest anechoic separation of the central echogenic complex (fat inside renal pelvis) in a horizontal axis. The stage of hydronephrosis of each kidney was recorded. The classification and scoring of schistosoma - related bladder lesions and measurement of congestive dilation of the renal pelvis were carried out using the method of WHO [1].

### Treatment

Twenty-three (23) children who identified with lesions immediately after the ultrasound investigation were on the spot treated with praziquantel tablets at 40 mg/kg body weight. An ultrasound check on the children was repeated after 3 and 6 months of treatment for lesion reversibility.

### Data analysis

Differences in proportion was analyzed using t-test and statistical significance was achieved if p < 0.05. Coefficient of determination was used to show the relationship between lesion and egg output.

## Results

Ultrasound examination showed pathological changes in the urinary tract of 23 (38.3%) out of the 60 children examined. Irregularities of the bladder inner surface was the most common changes identified in 25% of the pupils. Other changes included congestive changes in 6.7% of the individuals at the right pelvis and 1.7% at the left pelvis (see Table 1). These kidney changes were moderate. The urinary bladder intermediate score was 69 and upper urinary tract intermediate score was 30, thus giving a final *S. haematobium *score of 99.

**Table 1:** Distribution and types of lesion in the urinary tract.

**Organ and types of lesions**	**No**.	**%**	Score*
1. Urinary bladder (n = 60)			
Shape - Normal	37	61.7	-
- Distorted	23	38.3	23
2. Bladder wall (n = 60)			
Normal	37	61.7	-
Irregularity	15	25.5	30
Thickening	6	10	12
Mass	2	3.3	4
Pseudopolyp	0	0	-
Urinary bladder intermediate score			69
3. Kidney (n = 60)			
Right pelvis not dilated	56	93.3	-
Right pelvis moderately dilated	4	6.7	24
Marked hydronephrosis	0	0	-
Left pelvis not dilated	59	98.3	-
Left pelvis moderately dilated	1	1.7	6
Marked hydronephrosis	0	0	-
Upper urinary tract intermediate score			30
Final *S. haematobium *score			99

*WHO (2000) recommended mode of scoring.

n = number examined

Irregularities of the bladder wall was higher among male (60%) pupils when compared with females pupils (40%) (see Table 2). Also 4(66.7%) of the pupils with bladder thickness, were males while 2 (33.3%) were females. The two mass cases were males. Moderate kidney dilation was also observed in 4 males out of the 5 positive cases. The differences observed in lesion distribution among males and females was found to be significant (df = 6, p < 0.05) at 5% level. Of the 23 individuals associated with bladder wall lesion among which 5 were associated with kidney dilation, 19 (67.9%) belong to the age group 10-14. Others 21.4% and 10.7% belong to the age groups > 15 and 5-9 respectively (Table 3). The coefficient of determination showed that 98% of lesions was due to egg out put.

**Table 2:** Distribution of lesions by sex among the 23 with lesions.

**Lesion**	**No with lesions**	**Male**		**Female**	
		**No**	**%**	**No**	**%**
**Bladder wall** **Irregular**	15	9	60.0	6	40.0
**Thickness**	6	4	66.7	2	33.3
**Mass**	2	2	100	0	0
**Total**	**23**	**15**	**65.2**	**8**	**34.8**
					
**Kidney dilation**	5	4	80.0	1	20.0

**Table 3:** Comparison of intensity of infection and damage to urinary tract by age

**Age group/Egg count**	**Bladder wall irregularities**	**Bladder wall thickness**	**Bladder wall mass**	**Kidney dilation**	**Total/%**
5-9 yrs GM = 73.3	2	1	0	0	3(10.7)
					
10-14 yrs GM = 302.9	9	4	2	4	19(67.9)
					
> 15 yrs GM = 103.8	4	1	0	1	6(21.4)

Total	15	6	2	5	28(100)

GM = Geometric mean egg count.

Out of the 23 individuals with bladder wall lesions who were treated with praziquantel and monitored for six months, 16(69.6%) had their bladder wall healed after 3 months of treatment, while all except one was cured after six months of treatment with praziquantel (Table 4). The five cases of kidney dilation became normal after six months of treatment.

**Table 4:** Number of lesions after 6 months of treatment

**Months**	**Bladder wall lesions**					**Kidney dilation**	
	**No**.	**Irregular**	**Thickness**	**Mass**	**Normal**	**Dilation**	**Normal**
Initial	23	15	6	2	-	5	-
3	7	5	1	1	16	3	2
6	1	-	-	-	6	-	3
Cure rate					22(95.7%)		5(100%)

## Discussion

Irregularities of bladder wall seem to be a common lesion for individuals infected with *S. schistosomiasis *in Agulu community with highest score of 30. Bladder wall thickening and mass recorded low scores of 12 and 4 respectively. This result is similar to what was reported by [3] in West Madagascar and [4] in Zimbabwe. Mass and psuedoplyp which are outgrowth on bladder wall normally greater than 1 cm in size are not common in this area. This could be as a result of low endemicity of the disease in the study area or probably due to the fact that the people visit hospital for treatment as was reported by [5] in the same community. Only 5(83.3%) out of 60 children also had moderately dilated kidney. Reason could also be the same as above.

Higher percentage of males when compared with females had different types of lesions. This is statistically significant (df = 6, p < 0.05). This could be because more of the male pupils visit the Agulu lake (the source of infection) for recreational purposes like swimming than female pupil in same age group. This therefore brings them in contact with the schistosoma cercaria. They could have acquired more worm burden due to the length of time they stay inside water and thus have more lesions.

Pupil in the age group 10-14 years had the highest geometric mean egg output, and the highest number of lesions in their bladder and kidney. This shows that bladder and kidney lesions are associated with egg output. This is in agreement with Coopan *et al*. [6] finding in Natal Province of South Africa. Serieye *et al*. [3] and Hatz *et al*. [7] also observed that bladder and kidney lesions were associated with egg output. Both bladder and kidney lesions responded favorably to treatment with praziquantel. This agrees with the findings of [7] but however, in contrast to those of previous studies of [8] in which clearance of bladder lesions was not observed, which was probably partly due to the particular drugs in use at the time. Kidney lesions also resolved readily. Hatz *et al*. [7] had shown that even severe congestion of the kidneys showed considerable improvement six months after treatment. This is in contrast with the report of [9] who recorded delayed clearance of kidney lesions in coastal Kenya. This difference may be due to longer - standing disease or other transmission and host peculiarities, bacteria super infections or even a different reinfection pattern. A 12 months follow up study as reported by [10] indicates that pathlogical signs due to reinfection can develop within 6-12 months after treatment with the standard dose of praziquantel (40 mg/kg body wt). This information on the resolution of *S. haematobium *related lesions after treatment is crucial in defining treatment and retreatment schemes in relation to maintaining the lowest possible level of morbidity in a community [11]. However, since pathological signs can disappear spontaneously [12], more research after treatment against non-intervention areas is required. Earlier study by Ekwunife *et al *[5] in the same community has shown that community members have learnt to live with the disease, believing that as one gets older, the disease disappears without reference to the damage it caused to these organs. The photographs from ultrasonographic examination could be used for health education purposes in endemic communities. This will go a long way in the control and prevention of the disease in the affected community.

## Competing interests

The authors declare that they have no competing interests.

## Authors' contributions

OF participated in the designing of the study and performed the statistical. EC conceived the study, participated in the design, coordinated and helped to draft the manuscript. NO proof read the manuscript and helped in coordination. All authors participated in data collection, reading and approval of the final manuscript.
